# Hybrid Algorithms Based on Two Evolutionary Computations for Image Classification

**DOI:** 10.3390/biomimetics10080544

**Published:** 2025-08-19

**Authors:** Peiyang Wei, Rundong Zou, Jianhong Gan, Zhibin Li

**Affiliations:** 1 School of Computer Science and Technology, Chongqing University of Posts and Telecommunications, Chongqing 400065, China; 2 School of Software Engineering, Chengdu University of Information Technology, Chengdu 610225, China; aliwa8168@gmail.com (R.Z.); gjh@cuit.edu.cn (J.G.); lizhibin@cuit.edu.cn (Z.L.); 3 Chongqing Institute of Green and Intelligent Technology, Chinese Academy of Sciences, Chongqing 400714, China; 4 Software Automatic Generation and Intelligent Service Key Laboratory of Sichuan Province, Chengdu 610225, China; 5 Key Laboratory of Remote Sensing Application and Innovation, Chongqing 401147, China; 6 Dazhou Key Laboratory of Government Data Security, Sichuan University of Arts and Science, Dazhou 635000, China

**Keywords:** DenseNet-121, image classification, horned lizard optimization algorithm, giant armadillo optimization algorithm, hyperparameter optimization

## Abstract

Convolutional neural networks (CNNs) and their improved models (like DenseNet-121) have achieved significant results in image classification tasks. However, the performance of these models is still constrained by issues such as hyperparameter optimization and gradient vanishing and exploding. Owing to their unique exploration and exploitation capabilities, evolutionary algorithms offer new avenues for addressing these problems. Simultaneously, to prevent these algorithms from falling into a local optimum during the search process, this study designs a novel interpolation algorithm. To achieve better image classification performance, thus enhancing classification accuracy and boosting model stability, this paper utilizes a hybrid algorithm based on the horned lizard algorithm with quadratic interpolation and the giant armadillo optimization with Newton interpolation (HGAO) to optimize the hyperparameters of DenseNet-121. It is applied to five datasets spanning different domains. The learning rate and dropout rate have notable impacts on the outcomes of the DenseNet-121 model, which are chosen as the hyperparameters to be optimized. Experiments are conducted using the HGAO algorithm on five image datasets and compared with nine state-of-the-art algorithms. The performance of the model is evaluated based on accuracy, precision, recall, and F1-score metrics. The experimental results reveal that the combination of hyperparameters becomes more reasonable after optimization with the HGAO algorithm, thus providing a crucial improvement. In the comparative experiments, the accuracy of the image classification on the training set increased by up to 0.5%, with a maximum reduction in loss of 0.018. On the test set, the accuracy rose by 0.5%, and the loss decreased by 54 points. The HGAO algorithm provides an effective solution for optimizing the DenseNet-121 model. The designed method boosts classification accuracy and model stability, which also dramatically augments hyperparameter optimization effects and resolves gradient difficulties.

## 1. Introduction

Over the past decades, CNNs have made significant strides in image classification tasks and have been widely adopted in various image recognition and analysis applications. Conventional CNNs like AlexNet [1], GoogleNet [2], and ResNet [3] have excellent performance in different image classification tasks, thus driving continuous advancements in image processing technology. Meanwhile, emerging novel networks, like LSTM [4], ReNet [5], ViT [6], and DeiT [7], have tremendous potential, and can commendably address complex time-series data and cross-domain tasks [8,9,10,11].

Owing to the unique dense connectivity mechanism of DenseNet-121 [12], which has a higher classification performance on PlantVillage, we adopted this method for addressing the issue of image classification. However, its performance depends on the network design and hyperparameter settings. Most importantly, the learning rate and dropout rate play vital roles in the convergence speed and generalization ability of the model. Additionally, the learning rate determines the magnitude of the model weight adjustments. A high value results in oscillation and failure to converge, while a low value may cause a local optimum and slow convergence speed [2,12,13,14,15].

The latest research has further expanded the application scope of these networks [16,17,18,19,20]. Ma et al. developed a novel network called GoogLeNet-AL, which has better performance in lung cancer diagnoses, outperforming traditional GoogLeNet and other models [2]. Song et al. proposed a network model named DesTrans, which combines the advantages of DenseNet, ResNet, and Transformer to deal with medical images, and then applied it to multi-exposure, multi-focus, and infrared visible images [14]. Additionally, Chang et al. presented a wheat rust recognition method based on an improved DenseNet, which can effectively extract features from wheat leaf images [12]. Talukder et al. proposed a fine-tuned EfficientNet, which provides radiologists with an effective method for rapid and accurate COVID-19 diagnosis [16].

Although these models perform effectively in their respective applications, their hyperparameters are fixed values, which limits their full potential. Evolutionary algorithms have excellent potential in optimizing hyperparameters to achieve better results on specific tasks [1,4,21,22,23,24,25,26,27].

To address the above-mentioned issues, we propose an adaptive parameter optimization method, which was adopted to further enhance the performance of DenseNet-121 on image classification tasks. This method combines the following two different evolutionary algorithms: quadratic interpolation-based horned lizard optimization algorithm (QIHLOA) [8,17] and Newton interpolation-based giant armadillo optimization algorithm (NIGAO) [9,18], forming a hybrid algorithm called the “hybrid algorithm based on horned lizard optimization algorithm and giant armadillo optimization (HGAO)”. The proposed algorithm integrates the advantages of the HLOA and GAO algorithms, thus incorporating quadratic interpolation and Newton interpolation operations to enhance search capability and accuracy.

Evolutionary algorithms, biomimetic optimizers that simulate natural selection and biological evolution, improve deep learning performance via their bio-inspired properties. Notably, QIHLOA and NIGAO represent emerging biomimetic evolutionary methodologies that provide distinctive solutions for neural network-based image classification tasks. Their biomimetic traits crucially balance model complexity and generalization in deep learning. By emulating organisms’ adaptive survival strategies, these algorithms enable neural networks to dynamically tune topologies and optimize hyperparameters. This drives image classification from “data-driven” to “intelligently evolutionary”, unlocking the potential in few-shot learning and cross-domain recognition. Furthermore, the main contributions of this study are as follows:

(a) This study proposes an adaptive hyperparameter optimization method that combines QIHLOA and NIGAO algorithms. This algorithm is embedded into DenseNet-121, thus achieving efficient image classification. Notably, HGAO can quickly search for the optimal solution by adjusting the ratio of different weights β1 and β2, thereby improving the accuracy and speed of image classification.
(b) Four public datasets about image classification are gathered, which contain fields such as healthcare, agriculture, traditional medicine, remote sensing, and disaster management. Moreover, we make a self-developed traditional Chinese medicine image dataset. These datasets are sufficient for our experiments.
(c) Extensive experimental results indicate that the proposed method has high accuracy and efficiency in multi-domain image classification tasks. In particular, the proposed HGAO algorithm with DenseNet-121 outperforms several state-of-the-art algorithms—including HLOA, ESOA, PSO, and WOA—in multi-domain image classification tasks.

The rest of this paper is organized as follows: Section 2 presents the proposed method. Section 3 indicates the experimental results and analyzes the critical findings. Finally, Section 4 offers conclusions and future work.

## 2. Related Theoretical Description

### 2.1. DenseNet-121

**Figure 1:**
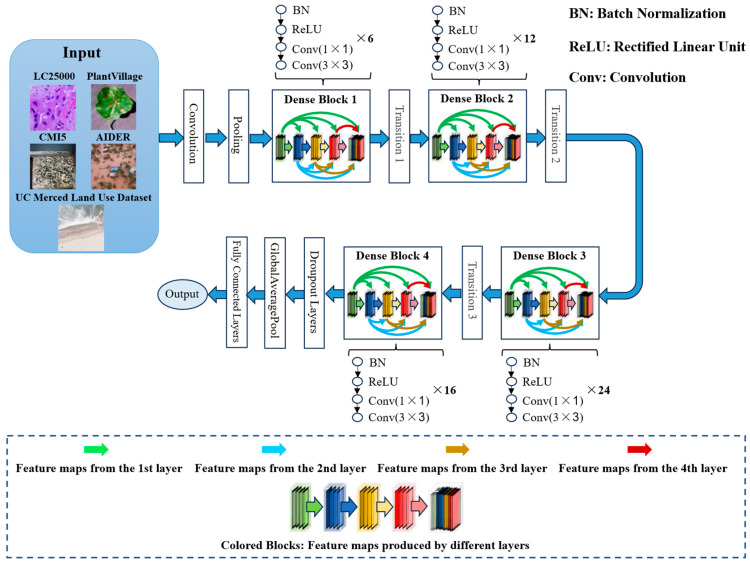
The structure of DenseNet-121, where each dense block consists of batch normalization (BN), ReLU, Conv(1 × 1), and Conv(3 × 3). Conv(1 × 1) represents a convolution operation with a kernel size of 1 × 1, and Conv(3 × 3) is a convolution operation with a kernel size of 3 × 3.

The major structure of DenseNet-121 is shown in Figure 1. This network consists of multiple dense blocks, transition layers, global average pooling layers, and fully connected layers. Each dense block is composed of several convolutional layers, which enhance the efficiency of information flow by adopting dense connections [5,6,7,28,29,30,31,32].

### 2.2. HLOA Algorithm

The horned lizard optimization algorithm (HLOA) is a novel bioinspired optimization algorithm [1] that simulates various defensive behaviors of the horned lizard, including crypsis, skin color changes, blood squirting, and escaping.

#### 2.2.1. Crypsis Behavior Strategy

Crypsis is a behavior that which organisms can simulate the characteristics of the environment to assimilate into their surroundings. This paper defines a color evaluation system, such as the Cartesian coordinate L∗a∗b system and the polar coordinate L∗C∗h system, which are used to calculate colors.

In the L∗a∗b system, L∗ represents luminosity, and a∗ and b∗ are chromaticity coordinates, which can be written as follows:

(1) $$\begin{array}{l} a*=\left\{\begin{array}{l} +a,\;indicates\;Red,\\ -a,\;indicates\;Green, \end{array}\right.\\ b*=\left\{\begin{array}{l} +b,\;indicates\;Yellow,\\ -b,\;indicates\;Blue, \end{array}\right. \end{array}$$

where L∗ defines brightness, C∗ specifies color intensity, and h∗ represents the hue angle.

(2) $$\begin{array}{l} {\vec{x}}_{i}\left(t+1\right)={\vec{x}}_{best}\left(t\right)+\left(\partial -\frac{\partial \cdot t}{Max_{iter}}\right)\times \\ \left(c_{1}\left(\sin\left({\vec{x}}_{r1}\left(t\right)\right)-\cos\left({\vec{x}}_{r2}\left(t\right)\right)\right)-{\left(-1\right)}^{\sigma }c_{2}\left(\cos\left({\vec{x}}_{r3}\left(t\right)\right)\right)-\sin\left({\vec{x}}_{r4}\left(t\right)\right)\right), \end{array}$$

where ${\vec{x}}_{i}\left(t+1\right)$ is the new individual position in the population space at generation *t* + 1; ${\vec{x}}_{best}\left(t\right)$ represents the best individual in generation *t*; and *r*1, *r*2, *r*3, and *r*4 are integer random numbers. $Max_{iter}$ is the maximum number of iterations, *σ* is a number, ∂ is set at 2, and *c*1 and *c*2 are random numbers, respectively.

#### 2.2.2. Skin Darkening or Lightening Strategy

Horned lizards can lighten or darken their skin, which depends on whether they need to reduce or increase the solar heat gain [1]. The skin color-changing strategy of the horned lizard can be represented as

(3) $$\begin{array}{ll} {\vec{x}}_{worst}\left(t\right) & ={\vec{x}}_{best}\left(t\right)+\frac{1}{2}Light_{1}\sin\left({\vec{x}}_{r1}\left(t\right)-{\vec{x}}_{r2}\left(t\right)\right)\\ & -{\left(-1\right)}^{\sigma }\frac{1}{2}Light_{2}\sin\left({\vec{x}}_{r3}\left(t\right)-{\vec{x}}_{r4}\left(t\right)\right), \end{array}$$

(4) $$\begin{array}{ll} {\vec{x}}_{worst}\left(t\right) & ={\vec{x}}_{best}\left(t\right)+\frac{1}{2}Dark_{1}\sin\left({\vec{x}}_{r1}\left(t\right)-{\vec{x}}_{r2}\left(t\right)\right)\\ & -{\left(-1\right)}^{\sigma }\frac{1}{2}Dark_{2}\sin\left({\vec{x}}_{r3}\left(t\right)-{\vec{x}}_{r4}\left(t\right)\right), \end{array}$$

where *Light*_1_, *Light*_2_, *Dark*_1_, and *Dark*_2_ are random numbers; ${\vec{x}}_{worst}\left(t\right)$ and ${\vec{x}}_{best}\left(t\right)$ are the worst and best individuals, respectively. *r*1, *r*2, *r*3, and *r*4 are integer random numbers; ${\vec{x}}_{r1}\left(t\right)$, ${\vec{x}}_{r1}\left(t\right)$, ${\vec{x}}_{r3}\left(t\right)$, and ${\vec{x}}_{r4}\left(t\right)$ are the individuals selected at positions *r*1, *r*2, *r*3, and *r*4. *σ* is a number.

#### 2.2.3. Blood-Squirting Strategy

Horned lizard defends themselves by spraying blood from their eyes. This behavior can be written as

(5) $$\begin{array}{ll} {\vec{x}}_{i}\left(t+1\right) & =\left(v_{0}\cos\left(\alpha \frac{t}{Max_{i}ter}+\varepsilon \right){\vec{x}}_{best}\left(t\right)\right)\\ & +\left(v_{0}\sin\left(\alpha -\frac{t}{Max_{i}ter}-g\right)+\varepsilon \right){\vec{x}}_{i}\left(t\right), \end{array}$$

where ${\vec{x}}_{i}\left(t+1\right)$, ${\vec{x}}_{i}\left(t\right)$ are the new individual position in the population at generation *t* + 1, *t*, respectively. ${\vec{x}}_{best}\left(t\right)$ is the best individual, $Max_{iter}$ is the maximum iterations, $v_{0}$ is set at 1 m/s, *α* is set at *π*/2, *ε* is set at 1 × 10^−6^, and *g* is the gravity of Earth.

#### 2.2.4. Move and Escape Strategy

In this strategy, the horned lizard moves quickly in the environment to evade predators.

(6) $${\vec{x}}_{i}\left(t+1\right)={\vec{x}}_{best}\left(t\right)+walk\left(\frac{1}{2}-\varepsilon \right){\vec{x}}_{i}\left(t\right),$$

where ${\vec{x}}_{i}\left(t+1\right)$ and ${\vec{x}}_{i}\left(t\right)$ are the position of a new individual in the population space at generation *t* + 1, *t*, respectively. ${\vec{x}}_{best}\left(t\right)$ is the best individual in generation *t*, and walk and *ε* are the random numbers, respectively, *σ*∈(0, 1).

#### 2.2.5. Melanophore-Stimulating Hormone Rate Strategy

The rapid color change observed on the skin of horned lizards is attributed to the effect of temperature on melanophore. The melanophore rate of horned lizard is defined as follows:

(7) $$melanophore\left(i\right)=\frac{F_{\max}-F\left(i\right)}{F_{\max}-F_{\min}},$$

where *F_min_* and *F_max_* are the best and worst fitness values in current generation *t*, respectively. *F*(*i*) is the current fitness value of the *i*-th individual. Thereafter, we obtain the following:

(8) $${\vec{x}}_{i}\left(t\right)={\vec{x}}_{best}\left(t\right)+\frac{1}{2}\left({\vec{x}}_{r1}\left(t\right)-{\left(-1\right)}^{\sigma }{\vec{x}}_{r2}\left(t\right)\right),$$

where ${\vec{x}}_{i}\left(t\right)$ is the current individual, ${\vec{x}}_{best}\left(t\right)$ is the best individual, and *r*1, *r*2 are integer random numbers, *σ*∈(0, 1).

### 2.3. Quadratic Interpolation Method

Quadratic interpolation is a local search operator [17] that can search for the optimal solution of the population in a known search space. Its learning rule is as follows:

(9) $$x_{h}=\frac{1}{2}\times \frac{\left(c_{h}^{2}-b_{h}^{2}\right)\times f\left(A\right)+\left(a_{h}^{2}-c_{h}^{2}\right)\times f\left(B\right)+\left(b_{h}^{2}-a_{h}^{2}\right)\times f\left(C\right)}{\left(c_{h}-b_{h}\right)\times f\left(A\right)+\left(a_{h}-c_{h}\right)\times f\left(B\right)+\left(b_{h}-a_{h}\right)\times f\left(C\right)},$$

where *A*, *B*, and *C* are three interpolation points; *f*(*A*), *f*(*B*), and *f*(*C*) are the fitness functions for *A*, *B*, and *C*, respectively.

To further enhance the search accuracy of the horned lizard optimization algorithm, we adopted a quadratic interpolation operator to modify its learning rule; its pseudocode is illustrated in Algorithm 1.
**Algorithm 1: QIHLOA****Input:** number of search agents *D*, population size *P*, maximum number of iterations *T* **Operation**
/* Initialization */1.**Initialize:** *D*, *P*, *T*
2.**Initialize:** Population initialization/* Training Starts */3.**for** *t* = 1 **to** |*T*|
4.  **for** *i* = 1 **to** |*P*|5. 6. 7. 8. 9. 10. 11. 12. 13. 14. 15. 16. 17. 18. 19. 20. 21. 22. 23. 24.  **if** Crypsis? **Then**   $\mathrm{Strategy}\;1:\;\mathrm{Update}\;{\vec{x}}_{i}\left(t+1\right)$ with (2).  **else**   **if** Flee? **Then**    $\mathrm{Strategy}\;4:\;\mathrm{Update}\;{\vec{x}}_{i}\left(t+1\right)$ with (6).   **else**    $\mathrm{Strategy}\;3:\;\mathrm{Compute}\;{\vec{x}}_{i}\left(t+1\right)$ with (5).   **end if**  **end if**     Strategy 2: Replace the worst population individual with (3) or (4).  **if** melanophore(*i*) ≤ 0.3 **Then**     $\mathrm{Strategy}\;5:\;\mathrm{Generate}\;a\;\mathrm{new}\;\mathrm{position}\;{\vec{x}}_{i}\left(t\right)$ with (8).  **end if**   $\mathrm{Calculate}\;F(i)\;\mathrm{for}\;\mathrm{the}\;\mathrm{new}\;\mathrm{individual}\;{\vec{x}}_{i}\left(t+1\right)$.   Use (9) to obtain a new individual *x_h_*.   $\mathrm{Update}\;{\vec{x}}_{best}\left(t\right)$ with (15).  **if** *F*(*i*) < *F_best_* **Then**   *F_best_* = *F*(*i*)   ${\vec{x}}_{best}\left(t\right)$$\;=\;{\vec{x}}_{i}\left(t\right)$  **end if**25. **end for**
26.**end for**
/* Operation Ending */$\text{Output:}\;{\vec{x}}_{best}\left(t\right)$, *F_best_*

### 2.4. GAO Algorithm

The GAO algorithm is a bionic metaheuristic algorithm [9] that simulates the natural behavior of wild giant armadillos.

#### 2.4.1. The Stage of Exploration

In the first phase of the GAO algorithm, the positions of population members in the problem-solving space are updated by the simulation of giant armadillos attacking termite mounds during hunting. Specially, the formula for updating the new position of each member in the population is as follows:

(10) $$X_{new}=X_{i,j}+r\cdot \left(STM_{i}-I\cdot X_{i}\right),$$

(11) $$X_{i}=\left\{\begin{array}{l} X_{new},F_{new}\leq F_{i},\\ X_{i},F_{new}>F_{i}, \end{array}\right.$$

where $STM_{i}$ is the selected termite mound of *i*-th giant armadillo; $X_{new}$ is the new position calculated for *i*-th giant armadillo based on the attack phase of the GAO algorithm; *F_new_* is the objective function value, *r*∈(0, 1); and *I* is random number.

#### 2.4.2. Digging in Termite Mounds

In the second phase of the GAO algorithm, the positions of population members in the solving space are updated based on the simulation of giant armadillos digging into termite mounds; its learning rule is as follows:

(12) $$X_{new}=X_{i}+\left(1-2\times r\right)\frac{ub-lb}{t},$$

(13) $$X_{i}=\left\{\begin{array}{l} X_{new},F_{new}\leq F_{i},\\ X_{i},F_{new}>F_{i}, \end{array}\right.$$

where *t* is the iteration. *ub* and *lb* are upper and lower bounds, respectively.

### 2.5. Newton Interpolation Method

Newton interpolation is a local optimization method [18], which is able to identify the optimal solution. By gradually adding data points, Newton interpolation can approximate the objective function, thereby improving computational accuracy. Moreover, its updating rule is written as follows:

(14) $$X_{b}=\frac{X_{last}+X_{i}}{2}-\frac{f^{\prime }\left(X_{last},X_{i}\right)}{2\cdot f^{\prime \prime }\left(X_{last},X_{i},X_{best}\right)},$$

(15) $$X_{i}=\left\{\begin{array}{l} X_{i},f\left(X_{i}\right)<f\left(X_{b}\right),\\ X_{b},f\left(X_{i}\right)\geq f\left(X_{b}\right), \end{array}\right.$$

where $f^{\prime }\left(\cdot \right)$ and $f^{\prime \prime }\left(\cdot \right)$ are the first derivative and second derivative, respectively. *X_last_* and *X_best_* are the best individuals from the last iteration and the global best individual, respectively. Furthermore, the major pseudocode of Newton’s interpolation optimization algorithm for a giant armadillo is shown in Algorithm 2.
**Algorithm 2: NIGAO****Input:** number of search agents *D*, population size *P*, maximum number of iterations *T* **Operation**

/* Initialization */1.**Initialize:** *D*, *P*, *T*
2.**Initialize:** Population initialization/* Training Starts */3.**for** *t* = 1 **to** |*T*|
4. **for** *i* = 1 **to** |*P*|5. 6. 7. 8. 9. 10. 11. 12. 13. 14. 15. 16. 17. 18.   **Phase 1: Attack on termite mounds**    Determine the termite mound set for the *i*-th population member *X_i_*    Randomly select the termite mound of *X_i_*.    Obtain a new position *X_new_* with (10).    Calculate the fitness of *X_new_* and update *X_i_* according to (11).   **Phase 2: Digging in termite mounds**    Update the position *X_new_* based on (12).    Calculate the fitness of *X_new_* and update *X_i_* according to (13).    Use (14) to obtain a new individual *X_b_*.    Update *X_best_* with (15).    **if**
*F_i_* < *F_best_*
**Then**:    *F_best_* = *F_i_*    *X_best_* = *X_i_*   **end if**19. **end for**20.**end for**
/* Operation Ending */**Output:** *X_best_*, *F_best_*

### 2.6. Association Optimization Algorithm (HGAO)

**Figure 2:**
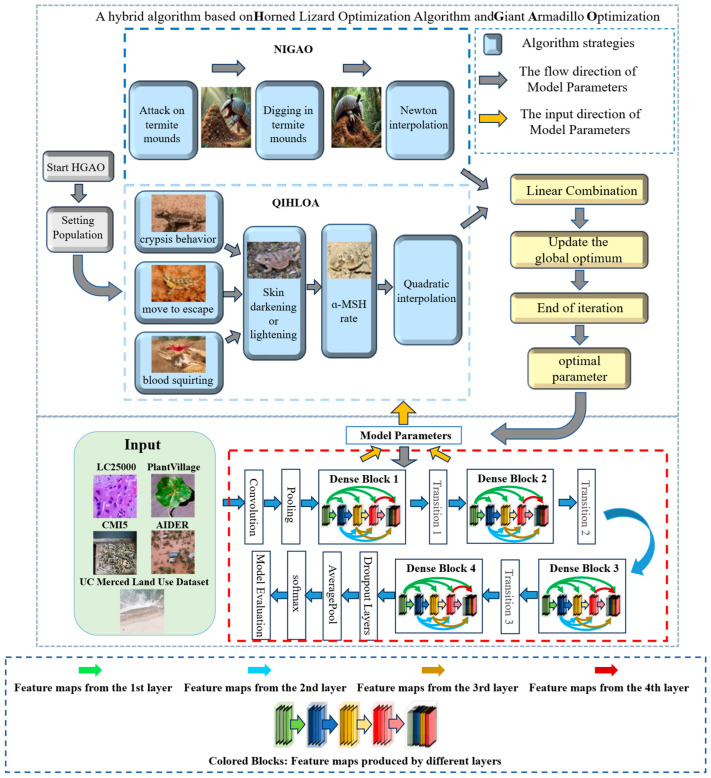
The overall framework of the HGAO algorithm.

In this section, we integrate the quadratic interpolation horned lizard optimization algorithm (QIHLOA) and the Newton interpolation giant armadillo algorithm (NIGAO) to form an HGAO algorithm. The overall framework of the HGAO algorithm is shown in Figure 2. It is important to note that both the QIHLOA and NIGAO algorithms produce the current optimal individual after each iteration. Moreover, new individuals are generated by using the following formula:

(16) $$X_{tnew}=\beta_{1}X_{i}+\beta_{2}Y_{i},$$

where *β*_1_ and *β*_2_ are the weights of the NIGAO and QIHLOA algorithms, respectively. *X_i_* and *Y_i_* represent the *i*-th population individual in the *t*-th iteration of NIGAO and QIHLOA algorithms, respectively. In particular, *X_tnew_* is the new individual after the integration.

Newton interpolation is widely applied in fields like engineering, physics, and scientific computing for tasks including curve fitting, simulations, and solving differential equations efficiently.

## 3. Experiments and Analyses

### 3.1. Experimental Setting

#### 3.1.1. Evaluation Metrics

In this section, we adopt several evaluation metrics, like accuracy, precision, and recall, to evaluate the effectiveness of the proposed algorithm [33,34,35,36,37,38,39,40,41,42], which are as follows:

(17) $$\begin{array}{l} Accuracy=\frac{TP+TN}{TP+TN+FP+FN},\\ Precision=\frac{TP}{TP+FP},\\ Recall=\frac{TP}{TP+FN},\\ F1\text{-}Score=\frac{2\times Precision\times Recall}{Precision+Recall}, \end{array}$$

where *TP* can be used to describe that the prediction is 1, and the actual value is 1, and thus the prediction is correct. *FP* indicates that the prediction is 1, but the actual value is 0, and thus the prediction is incorrect. *FN* describes that the [33,34,35,36,37,38,39,40,41,42] prediction is 0, but the actual value is 1, and thus the prediction is incorrect. *TN* presents that the prediction is 0, and the actual value is 0, and thus the prediction is correct.

#### 3.1.2. Datasets

**Figure 3:**
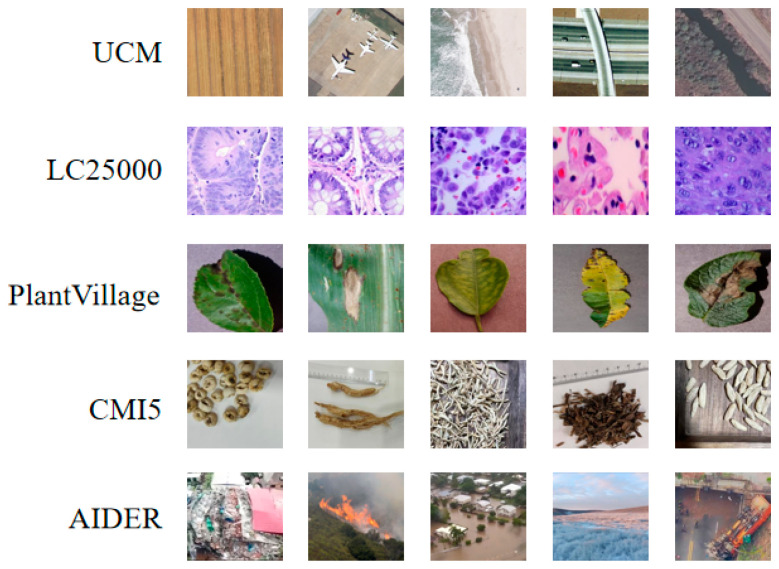
Representative sample images from the UCM, LC25000, PlantVillage, CMI5, and AIDER datasets.

We conducted experiments by using five different datasets, whose detailed information on the relevant datasets is shown in Table 1. Some example images from these datasets are illustrated in Figure 3 to provide a visual understanding of their characteristics.

(a) UC Merced Land Use Dataset (UCM) [22]: This is a standard dataset for remote sensing image classification, which is released by the University of California, Merced. This dataset contains 2100 high-resolution aerial images and has a size of 256 × 256 pixels. It consists of 17 different land use types, including farmland, forests, highways, parking lots, etc.
(b) LC25000 [20]: This consists of 25,000 color histopathological images, which can be used for classification tasks on lung and colon cancers. The dataset is evenly divided into five categories: lung adenocarcinoma, lung squamous cell carcinoma, benign lung tissue, colon adenocarcinoma, and benign colon tissue.
(c) PlantVillage [21]: This is an image dataset that contains 39 types of plant diseases, which is widely used in agricultural AI research. Moreover, this dataset includes 61,486 color images with a resolution of 256 × 256 pixels, which covers healthy and diseased leaf samples from common crops like vegetables, fruits, and grains.
(d) CMI5 (Chinese Medicine Identification 5 Dataset): We gathered this dataset, which contains images of traditional Chinese medicinal herbs. It consists of five categories: mint, fritillaria, honeysuckle, ophiopogon, and ginseng. Each category contains 2020 images; thus, it obtains a total of 10,100 images.
(e) AIDER (Aerial Image Dataset for Emergency Response applications) [23]: This is an aerial image dataset that is primarily used for classification tasks in emergency response scenarios. This dataset contains 5500 images with 5 categories, e.g., fire or smoke, floods, building collapse or debris, traffic accidents, and normal situations.

To fairly evaluate the proposed algorithm, 80% of this dataset was used for training; the remaining 20% was adopted for testing. Specifically, 10% of the training set was used for validation. Regarding input, all images were resized to 224 × 224 pixels. Thereafter, the batch size was set to 16, and stochastic gradient descent (SGD) was used as the optimizer. All experiments were conducted on a workstation equipped with two NVIDIA RTX A5000 GPUs and 128 GB of RAM (NVIDIA RTX A5000 GPU’s information: NVIDIA, Santa Clara, CA, USA; 128 GB of RAM’s information: Kingston, Fountain Valley, CA, USA. The Workstation was sourced from China).

### 3.2. Compared Algorithms

In this section, we compare the proposed HGAO algorithm with several state-of-the-art image classification algorithms to verify its experimental performance. The comparison algorithms are summarized as follows:

(a) M1: Horned Lizard Optimization Algorithm (HLOA) [8].
(b) M2: Giant Armadillo Optimization Algorithm (GAO) [9].
(c) M3: Particle Swarm Optimization (PSO) [29].
(d) M4: Egret Swarm Optimization Algorithm (ESOA) [30].
(e) M5: Black Widow Optimization (BWO) [31].
(f) M6: Transient Search Optimization Algorithm (TSO) [32].
(g) M7: Whale Optimization Algorithm (WOA) [33].
(h) M8: Catch fish Optimization Algorithm (CFOA) [34].
(i) M9: Goose Optimization Algorithm (GO) [35].

### 3.3. Combination Parameter Selection

In this section, we test two critical parameters, *β*_1_ and *β*_2_, of the HGAO algorithm based on DenseNet-121, which represent the weights of the NIGAO and QIHLOA optimization algorithms, respectively. The performance was compared with different algorithms, and then we evaluated five image classification datasets. Moreover, our goal is to validate the performance of HGAO under hyperparameters like the learning rate and dropout rate.

The learning rate is searched within the range of [0.00001, 0.1], and the dropout rate is within [0.1, 0.6]. To ensure fairness, all models were initialized with identical hyperparameters as follows: a population size of *P* = 30, a maximum of *T* = 10 optimization iterations, and a training process of 60 epochs.

We conducted five tests by changing the values of *β*_1_ and *β*_2_. *β*_1_ is set to 0.1, 0.3, 0.5, 0.7, and 0.9. *β*_2_ is set to 0.9, 0.7, 0.5, 0.3, and 0.1, respectively. These adjustments allow us to observe the effect of different weight configurations on the algorithm’s performance on various datasets. The specific results are listed in Table 2, Table 3, Table 4, Table 5 and Table 6.

In the experimental analysis, the performance of the HGAO algorithm on five datasets is unique; different combinations of *β*_1_ and *β*_2_ show the best performance. On the LC25000 and PlantVillage datasets, the combination (*β*_1_ = 0.3, *β*_2_ = 0.7) has the best performance, which achieves high test accuracy and low test loss. It is important to note that the moderate configuration of NIGAO weights combined with the high configuration of QIHLOA weights effectively prevents overfitting during optimization, which can maintain stable performance on complex data. On the UC Merced Land Use Dataset, the combination (*β*_1_ = 0.7, *β*_2_ = 0.3) obtains the best results; high NIGAO weights are beneficial for remote sensing image classification tasks. In particular, the optimization capabilities of NIGAO enhance the model’s classification performance in complex data. For the Chinese herbal medicine dataset, the combination (*β*_1_ = 0.1, *β*_2_ = 0.9) shows excellent results; it shows that high QIHLOA weights can utilize local features in high-dimensionality datasets to enhance the model’s generalization ability.

Finally, for the AIDER dataset, the combination (*β*_1_ = 0.3, *β*_2_ = 0.7) once again shows its advantages; it obtains the highest test accuracy and lowest test loss, which indicates that this combination is highly effective for classification tasks.

In summary, the experimental results from the five datasets show that different combinations (*β*_1_ and *β*_2_) have a significant effect on the performance of the HGAO algorithm on various datasets. Among them, the configuration *β*_1_ = 0.3, *β*_2_ = 0.7 obtains the best performance on most datasets, which can balance the weights of NIGAO and QIHLOA, thus leading to excellent performance in both the training and testing phases.

**Figure 4:**
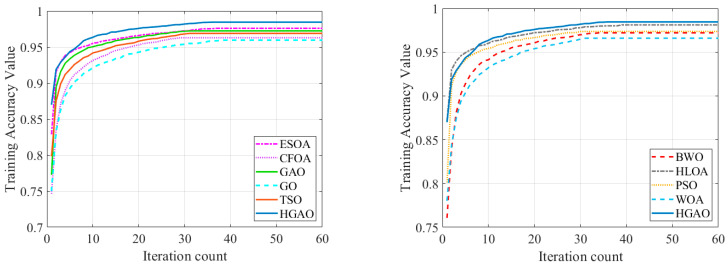
The training accuracy on LC25000.

The average accuracy of HGAO and nine compared algorithms during the computation process is displayed in Figure 4. Due to the density of the 10 lines, the results are split into two graphs; the convergence graphs for the remaining datasets are accessed by a hyperlink, which is in the Data Availability Statement section. In the early epochs, the HLOA algorithm outperforms the HGAO algorithm, which could find an initial solution faster. However, HGAO gradually surpassed HLOA with its optimization strategy. As training progressed, it showed stronger global optimization capabilities. The advantage of HGAO depends on its ability to effectively escape local optimum, thus obtaining higher accuracy and lower loss in the later stages of training.

Table 7, Table 8, Table 9, Table 10 and Table 11 present the performance of various compared algorithms on five datasets. In these tables, the HGAO algorithm obtains the lowest and best training and testing losses on these datasets. The convergence of the training process in Figure 4 illustrates the accuracy of HGAO on the training and testing sets. For the LC25000 dataset, HGAO achieves excellent performance, obtaining a test accuracy of 99.93% and a test loss of 0.014. Its precision, recall, and F1-score can reach 0.95, which outperforms other compared algorithms.

Although HLOA and GAO perform splendidly during training, the performance of HGAO in the testing phase is 0.52% higher than the accuracy of HLOA, which achieves a better balance between training and testing performance. For the other four datasets, i.e., PlantVillage, UC Merced Land Use Dataset, Chinese Medicinal Materials, and AIDER, HGAO also obtains outstanding performance. In particular, it achieves a test accuracy of 99.06% and a test loss of 0.0333 on the CMI5 dataset. These findings indicate that the HGAO algorithm with accurate hyperparameters can maintain high accuracy during the training process, and it also significantly improves generalization during the testing process, thus showing its exceptional performance in various image classification tasks.

## 4. Conclusions

This paper proposes an innovative HGAO algorithm to enhance the accuracy and recognition performance in multi-domain image classification tasks. It combines two different evolutionary algorithm strategies, thus enhancing the local search capability and global search precision by integrating quadratic interpolation and Newton interpolation. A novel linear combination strategy is also employed to enhance overall performance. To validate the effectiveness of this algorithm, extensive experiments were conducted on five multi-domain image datasets.

Extensive experiments conducted on five diverse image classification datasets—spanning remote sensing, medical imaging, plant disease detection, traditional Chinese medicine, and emergency response—demonstrate that HGAO consistently outperforms multiple baseline optimization methods. In particular, it achieves superior classification accuracy, improved stability, and stronger global optimization capability. These results validate the robustness, adaptability, and strong generalization ability of the proposed algorithm across heterogeneous data domains.

In the future, we plan to further expand the HGAO algorithm by incorporating various advanced evolutionary algorithms and optimization strategies, thus exploring its potential applications in other complex optimization issues. Additionally, we plan to investigate how adaptively adjusting the weight parameters of the combination strategy for different types of tasks can further enhance the algorithm’s generalization ability and robustness to promote the practical application of the HGAO algorithm in multi-domain optimization problems.

## Figures and Tables

**Table 1:** Detailed information for relevant datasets.

Dataset	Categories	Sizes (M)	Image Count
UC Merced Land Use Data	21	418 MB	2100
LC25000	5	1.75 GB	25,000
AIDER	5	263 MB	6433
PlantVillage	39	898 MB	61,486
Chinese Medicinal Materials	5	117 MB	10,100

**Table 2:** Experimental results on LC25000.

*β*_1_	*β*_2_	Training Accuracy	Training Loss	Test Accuracy	Test Loss	Precision	Recall	F1-Score
0.1	0.9	0.992	0.029	0.950	0.153	0.950	0.950	0.950
0.3	0.7	0.993	0.014	0.951	0.140	0.950	0.950	0.950
0.5	0.5	0.983	0.054	0.948	0.189	0.950	0.950	0.950
0.7	0.3	0.989	0.039	0.949	0.223	0.950	0.950	0.950
0.9	0.1	0.985	0.046	0.942	0.206	0.940	0.940	0.940

**Table 3:** Experimental results on PlantVillage.

*β*_1_	*β*_2_	Training Accuracy	Training Loss	Test Accuracy	Test Loss	Precision	Recall	F1-Score
0.1	0.9	0.998	0.006	0.969	0.104	0.970	0.970	0.970
0.3	0.7	0.998	0.004	0.972	0.092	0.970	0.970	0.970
0.5	0.5	0.995	0.016	0.969	0.099	0.970	0.970	0.970
0.7	0.3	0.998	0.006	0.97	0.104	0.970	0.970	0.970
0.9	0.1	0.997	0.084	0.967	0.111	0.970	0.970	0.970

**Table 4:** Experimental results on UC Merced land use dataset.

*β*_1_	*β*_2_	Training Accuracy	Training Loss	Test Accuracy	Test Loss	Precision	Recall	F1-Score
0.1	0.9	0.999	0.005	0.991	0.033	0.990	0.990	0.990
0.3	0.7	0.998	0.008	0.985	0.052	0.990	0.990	0.990
0.5	0.5	0.998	0.007	0.987	0.390	0.990	0.990	0.990
0.7	0.3	0.997	0.009	0.980	0.066	0.980	0.980	0.980
0.9	0.1	0.996	0.010	0.979	0.069	0.980	0.980	0.980

**Table 5:** Experimental results on CMI5.

*β*_1_	*β*_2_	Training Accuracy	Training Loss	Test Accuracy	Test Loss	Precision	Recall	F1-Score
0.1	0.9	0.999	0.005	0.990	0.033	0.990	0.990	0.990
0.3	0.7	0.998	0.008	0.985	0.052	0.990	0.990	0.990
0.5	0.5	0.999	0.006	0.987	0.039	0.990	0.990	0.990
0.7	0.3	0.997	0.009	0.980	0.066	0.980	0.980	0.980
0.9	0.1	0.996	0.009	0.979	0.069	0.980	0.980	0.980

**Table 6:** Experimental results on AIDER.

*β*_1_	*β*_2_	Training Accuracy	Training Loss	Test Accuracy	Test Loss	Precision	Recall	F1-Score
0.1	0.9	0.991	0.021	0.910	0.367	0.910	0.910	0.910
0.3	0.7	0.998	0.004	0.917	0.339	0.920	0.920	0.920
0.5	0.5	0.994	0.010	0.909	0.364	0.910	0.910	0.910
0.7	0.3	0.994	0.008	0.914	0.349	0.910	0.910	0.910
0.9	0.1	0.992	0.016	0.905	0.386	0.910	0.900	0.900

**Table 7:** Algorithm comparison on LC25000.

Compared Algorithm	Training Accuracy	Training Loss	Test Accuracy	Test Loss	Precision	Recall	F1-Score
HLOA	0.988	0.036	0.947	0.194	**0.950**	**0.950**	**0.950**
GAO	0.986	0.033	0.936	0.220	0.940	0.940	0.940
PSO	0.984	0.035	0.934	0.230	0.930	0.930	0.930
ESOA	0.985	0.032	0.932	0.250	0.930	0.930	0.930
BWO	0.981	0.082	0.918	0.254	0.920	0.920	0.920
TSO	0.982	0.048	0.925	0.240	0.930	0.930	0.930
WOA	0.979	0.061	0.903	0.313	0.900	0.900	0.900
CFOA	0.982	0.058	0.896	0.339	0.900	0.900	0.900
GO	0.976	0.063	0.923	0.258	0.920	0.920	0.920
HGAO	0.993	0.014	0.951	0.140	0.950	0.950	0.950

**Table 8:** Algorithm comparison on PlantVillage.

Compared Algorithm	Training Accuracy	Training Loss	Test Accuracy	Test Loss	Precision	Recall	F1-Score
HLOA	0.997	0.009	0.970	0.102	0.970	0.970	0.970
GAO	0.996	0.011	0.969	0.105	0.970	0.970	0.970
PSO	0.991	0.026	0.964	0.112	0.960	0.960	0.960
ESOA	0.994	0.017	0.965	0.111	0.970	0.960	0.960
BWO	0.994	0.019	0.964	0.113	0.960	0.960	0.960
TSO	0.989	0.030	0.961	0.135	0.960	0.960	0.960
WOA	0.988	0.034	0.967	0.106	0.970	0.970	0.970
CFOA	0.991	0.022	0.965	0.109	0.960	0.960	0.960
GO	0.989	0.028	0.961	0.135	0.960	0.960	0.960
HGAO	0.998	0.004	0.972	0.092	0.970	0.970	0.970

**Table 9:** Algorithm comparison on UC Merced land use dataset.

Compared Algorithm	Training Accuracy	Training Loss	Test Accuracy	Test Loss	Precision	Recall	F1-Score
HLOA	0.995	0.016	0.921	0.252	0.920	0.920	0.920
GAO	0.991	0.039	0.917	0.384	0.920	0.920	0.920
PSO	0.974	0.1888	0.911	0.451	0.910	0.910	0.910
ESOA	0.993	0.028	0.882	0.620	0.880	0.880	0.880
BWO	0.984	0.104	0.890	0.575	0.890	0.890	0.890
TSO	0.974	0.172	0.871	0.670	0.870	0.870	0.870
WOA	0.966	0.241	0.852	0.783	0.850	0.850	0.850
CFOA	0.985	0.101	0.894	0.576	0.890	0.890	0.890
GO	0.978	0.136	0.898	0.521	0.900	0.900	0.900
HGAO	0.998	0.008	0.923	0.224	0.920	0.920	0.920

**Table 10:** Algorithm comparison on CMI5.

Compared Algorithm	Training Accuracy	Training Loss	Test Accuracy	Test Loss	Precision	Recall	F1-Score
HLOA	0.997	0.008	0.987	0.446	0.990	0.990	0.990
GAO	0.994	0.030	0.973	0.092	0.970	0.970	0.970
PSO	0.991	0.064	0.957	0.131	0.960	0.960	0.960
ESOA	0.991	0.050	0.962	0.108	0.960	0.960	0.960
BWO	0.989	0.054	0.972	0.085	0.970	0.970	0.970
TSO	0.985	0.077	0.957	0.132	0.960	0.960	0.960
WOA	0.988	0.068	0.966	0.108	0.970	0.970	0.970
CFOA	0.985	0.073	0.970	0.080	0.970	0.970	0.970
GO	0.987	0.095	0.944	0.168	0.940	0.940	0.940
HGAO	0.999	0.005	0.991	0.033	0.990	0.990	0.990

**Table 11:** Algorithm comparison on AIDER.

Compared Algorithm	Training Accuracy	Training Loss	Test Accuracy	Test Loss	Precision	Recall	F1-Score
HLOA	0.994	0.010	0.912	0.352	0.910	0.910	0.910
GAO	0.992	0.014	0.908	0.374	0.910	0.910	0.910
PSO	0.987	0.039	0.896	0.556	0.900	0.900	0.900
ESOA	0.987	0.035	0.904	0.392	0.900	0.900	0.900
BWO	0.978	0.063	0.872	0.613	0.870	0.870	0.870
TSO	0.978	0.068	0.893	0.555	0.890	0.890	0.890
WOA	0.983	0.057	0.870	0.745	0.870	0.870	0.870
CFOA	0.986	0.044	0.887	0.649	0.890	0.890	0.890
GO	0.977	0.061	0.873	0.687	0.870	0.870	0.870
HGAO	0.998	0.004	0.917	0.339	0.920	0.920	0.920

## Data Availability

We collect five different datasets. CMI5 is self-constructed, which can be obtained from the following link: https://github.com/aliwa8168/HGAO/tree/main/Datasets/Chinese%20herbal%20medicine%20Datasets (accessed on 16 August 2025). The remaining four datasets can be obtained from relevant literature.
